# High Vaccination Coverage among Children during Influenza A(H1N1)pdm09 as a Potential Factor of Herd Immunity

**DOI:** 10.3390/ijerph13101017

**Published:** 2016-10-17

**Authors:** Toshihiko Matsuoka, Tomoki Sato, Tomoyuki Akita, Jiturou Yanagida, Hiroki Ohge, Masao Kuwabara, Junko Tanaka

**Affiliations:** 1 Department of Epidemiology, Infectious Disease Control and Prevention, Institute of Biomedical and Health Sciences, Hiroshima University, Hiroshima 734-8551, Japan; d133416@hiroshima-u.ac.jp (T.M.); kounu-satou@outlook.com (T.S.); tomo-akita@hiroshima-u.ac.jp (T.A.); 2 Hiroshima City Funairi Citizens Hospital, Hiroshima 730-0844, Japan; yanayana@ruby.ocn.ne.jp; 3 Department of Infectious Diseases, Hiroshima University Hospital, Hiroshima 734-8551, Japan; ohge@hiroshima-u.ac.jp; 4 Hiroshima Prefectural Center for Disease Control and Prevention, Hiroshima 734-0007, Japan; ba_ramasa@yahoo.co.jp

**Keywords:** Influenza A(H1N1)pdm09, vaccination, herd immunity, expansion of infection, pandemic

## Abstract

The objective of this study was to identify factors related to the expansion of infection and prevention of influenza A(H1N1)pdm09. A retrospective non-randomized cohort study (from June 2009 to May 2010) on influenza A(H1N1)pdm09 was conducted in a sample of residents from Hiroshima Prefecture, Japan. The cumulative incidence of the influenza A(H1N1)pdm09 and the pandemic vaccine effectiveness (VE) were estimated. The response rate was 53.5% (178,669/333,892). Overall, the odds ratio of non-vaccinated group to vaccinated group for cumulative incidence of influenza A(H1N1)pdm09 was 2.18 (95% confidence interval (CI): 2.13–2.23) and the VE was 43.9% (CI: 42.8–44.9). The expansion of infection, indicating the power of transmission from infected person to susceptible person, was high in the 7–15 years age groups in each area. In conclusion, results from this survey suggested that schoolchildren-based vaccination rate participates in determining the level of herd immunity to influenza and children might be the drivers of influenza transmission. For future pandemic preparedness, vaccination of schoolchildren may help to prevent disease transmission during influenza outbreak.

## 1. Introduction

In the 20th century, there were three influenza pandemics: the 1918–1919 Spanish flu (A/H1N1), the 1957–1958 Asian flu (A/H2N2), and the 1968–1969 Hong Kong flu (A/H3N2) [1]. In the early 21st century, influenza A(H1N1)pdm09 began in Mexico and the USA in April 2009 and rapidly spread globally. In Japan, the first case of H1N1 infection was confirmed in May 2009, after which the disease spread quickly across the nation. Between May 2009 and August 2010, influenza A(H1N1)pdm09 affected approximately 21 million people and caused 203 deaths among 127 million people in the country [2]. Under abnormal situations such as the 2009 influenza pandemic, it would be beneficial to identify the route of infection, analyze pandemic dynamics and build a vaccination strategy to safeguard against the pandemic. Avoiding infection has an impact on medical and economic burden [3]. Influenza vaccine effectiveness is not always evident [4,5]. Herd immunity refers to the resistance of a community against a given disease to which a large proportion of individual in the community are immune [6]. Nearly 80% to 90% immune population are required to establish herd immunity against influenza viruses [7]. Japan experienced low excess mortality from influenza when the country adopted the strategy to administer seasonal influenza vaccines to schoolchildren in the 1970s and 1980s, but excess mortality increased in the 1990s, when group vaccinations were halted [8,9,10]. The Japanese government started the administration of the pandemic monovalent vaccine in November 2009 in a priority phased manner to effectively address the 2009 pandemic influenza. The priority was given to pregnant women and patients with diseases that could be aggravated by influenza A(H1N1)pdm09 [11]. To provide information for planning preventative measures against influenza infection, it is crucial to evaluate pandemic influenza transmission among humans. However, there are a few studies that have addressed the relationship between pandemic dynamics and preventative action including vaccine [12] and hand hygiene [13,14].

In an effort to identify factors related to the prevention of H1N1 influenza infection in Hiroshima Prefecture, we analyzed existing data from a survey (*n* = 176,113) on the 2009 pandemic H1N1 influenza infection carried out by the Regional Health Care Council of Hiroshima Prefecture. We considered strategies against the pandemic, the vaccine effectiveness (VE), and the associations between herd immunity and cumulative incidence. We also estimated the expansion of H1N1 influenza infection using a mathematical model, and considered the factors that affect the dynamics of a pandemic using survey skills.

## 2. Materials and Methods

### 2.1. Study Design and Participants

**Figure 1:**
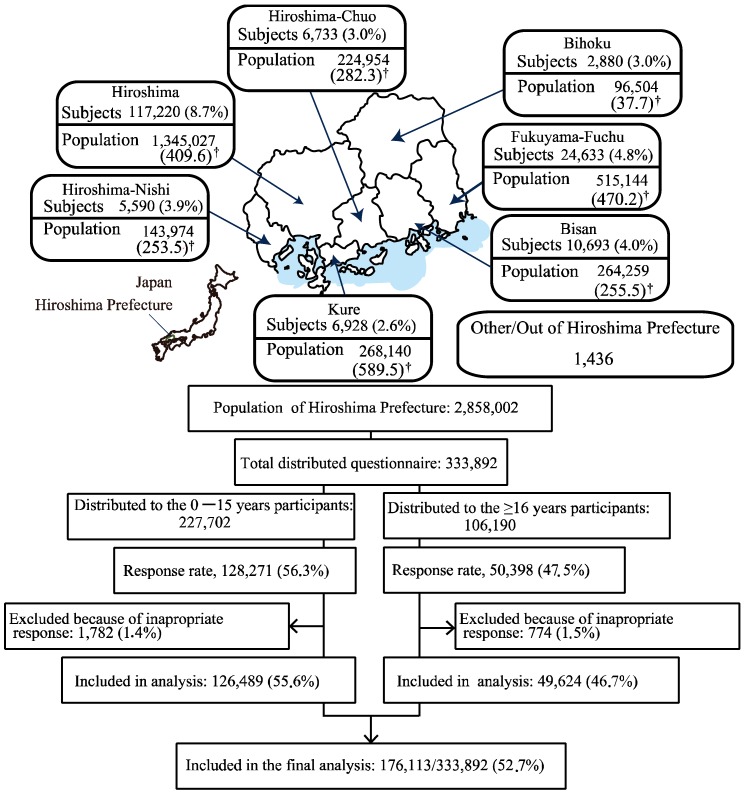
Map of Hiroshima Prefecture by place of living and flow chart of the study population. Hiroshima Prefecture is located in the west of Japan. The prefecture is divided into seven medical administration areas. Subjects living outside of Hiroshima Prefecture were only used for logistic regression analysis. ^†^ refers to population density (people per km^2^).

To identify potential factors of herd immunity during influenza A(H1N1)pdm09, between July and October 2010, the Regional Health Care Council of Hiroshima Prefecture carried out a retrospective non-randomized cohort study (from June 2009 to May 2010) on the 2009 H1N1 influenza infection among 176,113 individuals (82,738 males, and 93,375 females). The survey included schoolchildren (belonging to kindergartens, primary schools, middle schools, and high schools), community residents and workers from factories/companies. The survey took place in Hiroshima Prefecture located in the Western region of Japan. In March 2009, the prefecture of Hiroshima was inhabited by 2,858,002 people [15] and consisted of seven medical areas (Figure 1, Table 1).

All individuals who lived or worked in Hiroshima Prefecture were considered as potential participants. They were contacted through their supervisors (workers in factories), medical doctors (elderly), and parents/guardians (children); invitation letters containing a brief explanation of the survey were mailed to all the supervisors. Those interested in the survey attended a briefing meeting held at a local community center. Participation was on a voluntary basis and being present in Hiroshima Prefecture during the survey was the inclusion criteria. Living outside of Hiroshima Prefecture was the exclusion criteria except for logistic regression analysis.

### 2.2. Questionnaires

Participants were asked to fill out an anonymous, self-administered questionnaire on paper, with younger participants (0–15 years) being helped by their parents or legal guardians.

We used two similar questionnaires (originally written in Japanese language; Supplementary File), one for participants aged 0–15 years and another one for those over 16 years. The two questionnaires were designed by the Regional Health Care Council of Hiroshima Prefecture and consisted of fourteen items each (open and multiple-choice answers) divided into two parts. The first part included three items for recording participants’ characteristics such as living area, age or age group and gender. The second part specifically addressed beliefs and information sources related to the 2009 influenza outbreak and the experience of infection with influenza A(H1N1)pdm09. The following eight questions provide a generic explanation of the content of the eleven items used in the second part of each questionnaire:

- What was the most helpful information about influenza A(H1N1)pdm09?
- Have you/your child had influenza A(H1N1)pdm09 infection?
- Did a doctor confirm your influenza A(H1N1)pdm09 infection between June 2009 to May 2010? Which month was it?
- How many people are living together in your family?
- Which preventative action did you take against influenza A(H1N1)pdm09?
- Have you/your child been vaccinated against influenza A(H1N1)pdm09?
- What is the reason for being vaccinated?
- What is the reason for not being vaccinated?

For feasibility purposes, the participants were divided into two groups: 0–15 years and 16 years or above. However, in the analysis, more than two age-specific groups were used. Positive case of influenza A(H1N1)pdm09 was defined as any positive answer to the following question: “Did a doctor confirm your influenza A(H1N1)pdm09 infection?”

### 2.3. Statistical Analysis

We calculated period prevalence per month (from June 2009 to May 2010), vaccination rates, overall cumulative incidence, cumulative incidence by vaccine status and odds ratio (OR), VE, and expansion of infection. In addition, we used logistic regression models to clarify the relationship between these estimated indicators and preventative measures.

#### 2.3.1. Period Prevalence by Month (June 2009 to May 2010)

To estimate period prevalence, the number of participants affected per month was stratified by gender, medical administration area and age group (0; 1–3; 4–6; 7–9; 10–12; 13–15; 16–19; 20–29; 30–39; 40–49; 50–59; 60–69; 70–79; and ≥80 years).

#### 2.3.2. Vaccination rates (from November 2009 to May 2010)

Vaccination rate was obtained using:

$$\mathrm{Vaccination}\;\mathrm{rate}\;=\;\frac{\mathrm{Number}\;\mathrm{of}\;\mathrm{participants}\;\mathrm{vaccinated}}{\left(\mathrm{Number}\;\mathrm{of}\;\mathrm{participants}\right)\;-\;\left(\mathrm{Number}\;\mathrm{of}\;\mathrm{participants}\;\mathrm{infected}\;\mathrm{up}\;\mathrm{to}\;\mathrm{October}\;2009\right)}\times \;100$$

#### 2.3.3. Overall Cumulative Incidence (from November 2009 to May 2010)

Overall cumulative influenza incidence was estimated using the following formula:

$$\mathrm{Overall}\;\mathrm{cumulative}\;\mathrm{incidence}\;=\;\frac{\mathrm{Number}\;\mathrm{of}\;\mathrm{infected}\;\mathrm{participants}\;\mathrm{from}\;\mathrm{November}\;2009\;\mathrm{to}\;\mathrm{May}\;2010}{\mathrm{Number}\;\mathrm{of}\;\mathrm{participants}}\;\times \;100$$

#### 2.3.4. Cumulative Incidence by Vaccine Status and Odds Ratio (November 2009 to May 2010)

Participants who were infected with influenza A(H1N1)pdm09 before implementation of influenza A(H1N1)pdm09 mass vaccination were excluded (because the vaccination campaign was launched in November 2009 [11]). We further excluded those with missing vaccination data. They were placed into one of the twelve age groups (0; 1–3; 4–6; 7–9; 10–12; 13–15; 16–19; 20–29; 30–39; 40–49; 50–59; and ≥60 years). The following formulae were used:

$$\mathrm{Cumulative}\;\mathrm{incidence}\;\mathrm{for}\;\mathrm{vaccinated}\;\mathrm{group}\;=\;\frac{\mathrm{Number}\;\mathrm{of}\;\mathrm{infected}\;\mathrm{participants}\;\mathrm{in}\;\mathrm{vaccinated}\;\mathrm{group}\;\mathrm{from}\;\mathrm{November}\;2009\;\mathrm{to}\;\mathrm{May}\;2010}{\mathrm{Number}\;\mathrm{of}\;\mathrm{vaccinated}\;\mathrm{participants}\;}\;\times \;100$$

Odds ratios of non-vaccinated group to vaccinated group for cumulative incidence of influenza A(H1N1)pdm09 were estimated using:

$$\mathrm{OR}\;\mathrm{for}\;\mathrm{cumulative}\;\mathrm{incidence}\;=\;\frac{\frac{\left(\mathrm{Number}\;\mathrm{of}\;\mathrm{infected}\;\mathrm{participants}\;\mathrm{in}\;\mathrm{non}-\mathrm{vaccinated}\;\mathrm{group}\right)}{\left(\mathrm{Number}\;\mathrm{of}\;\mathrm{non}-\mathrm{infected}\;\mathrm{participants}\;\mathrm{in}\;\mathrm{non}-\mathrm{vaccinated}\;\mathrm{group}\right)}}{\frac{\left(\text{Number of infected participants in vaccinated group}\right)}{\left(\text{Number of non}-\text{infected participants in vaccinated group}\right)}}$$

Furthermore, differences in ORs stratified by medical administration area, age group, and gender were examined using the Breslow-Day test.

#### 2.3.5. Vaccine Effectiveness (from November 2009 to May 2010)

We estimated VE as follows:

$$\mathrm{VE}\;=\;\frac{\begin{array}{c} \left(\mathrm{Cumulative}\;\mathrm{incidence}\;\mathrm{of}\;\mathrm{non}-\mathrm{vaccinated}\;\mathrm{group}\right)\\ \;-\;\left(\mathrm{Cumulative}\;\mathrm{incidence}\;\mathrm{of}\;\mathrm{vaccinated}\;\mathrm{group}\right)\; \end{array}}{\left(\mathrm{Cumulative}\;\mathrm{incidence}\;\mathrm{of}\;\mathrm{non}-\mathrm{vaccinated}\;\mathrm{group}\right)}$$

#### 2.3.6. Correlation between Vaccination Rates and Cumulative Incidence

In order to assess the impact of age-specific vaccination program on herd immunity, we explored the correlation between age-specific vaccination rates and cumulative incidence for the overall area. In other words, we confirmed the age group that should be vaccinated to reduce cumulative incidence in the area.

#### 2.3.7. Logistic Regression Analysis for Preventing Infection

Logistic regression analysis was performed with the infection with influenza A(H1N1)pdm09 as the objective variable for all participants and subgroups (participants aged 0–15 years, and those aged ≥16 years). Gender, age group, medical administration area, source of helpful information related to the influenza A(H1N1)pdm09 outbreak, and preventative measures against influenza were the explanatory variables. The baseline group was defined as gender “female”, medical administration area “Hiroshima”, age group “4–6 years” (for all participants and those aged 0–15 years) or “30–39 years” (for participants ≥16 years), “no information was helpful”, and “did not pay attention to anything”.

#### 2.3.8. Expansion of Infection

We defined expansion of infection as a probability of infection in sensitive persons when they had contact with infected persons. Participants who did not provide valid responses on the gender, age and medical administration area were excluded. Therefore, a total of 175,345 responses were available to estimate the expansion of infection. Multi-type epidemic model was used to determine the expansion of infection per group [16]. The groups were stratified by gender, age group, and medical administration area. Parameters of the model were estimated using the maximum likelihood method.

Furthermore, multiple linear regression analysis was used to assess the relationship between the expansion of infection and the studied parameters. This model included the following explanatory variables: medical administration area, age groups, gender, household size, and preventive measures (vaccination, thorough gargling/hand hygiene, use of a mask, cough etiquette, avoidance of being in crowds, and getting plenty of rest). The 4–6 year group was considered as the baseline age group, and Hiroshima was considered as the baseline medical administration area.

The significant level was set as *p* < 0.05. JMP version 9 (SAS Institute Inc., Cary, NC, USA) was used for analyses.

### 2.4. Ethics Approval and Consent to Participate

This study was approved by the ethics committee for epidemiological research at Hiroshima University (epidemiology No. 411). Specifically, the informed consent for the survey was also included in the application form to the ethics committee. Participants who submitted the questionnaire were considered to be in agreement with it. After submitting the questionnaire, participants could not opt out because the survey was performed anonymously.

## 3. Results

### 3.1. Participants’ Characteristics

A total of 333,892 questionnaires were distributed, and responses were received from 178,669 participants (response rate, 53.5%). There were 1.4% surveys (2556/178,669) excluded from analysis because of missing data. Therefore, the final sample included 176,113 (98.6%) participants. This is equivalent to 6.2% of the population of Hiroshima Prefecture [15].

The majority (71.8%) of the participants were aged 0–15 years. Comparatively, there were relatively more females (*n* = 93,375; 53.0%) than males (*n* = 82,738; 47.0%).

### 3.2. Vaccination Rates

As presented in Table 2, the overall vaccination rate against influenza A(H1N1)pdm09 was 36.5%. There were significant differences in vaccination rates regarding gender, age, and area. As for age-specific vaccination rates, participants aged ≥70 years and those aged 1–6 years were found with higher rates as compared with other age groups.

### 3.3. Period Prevalence

**Figure 2:**
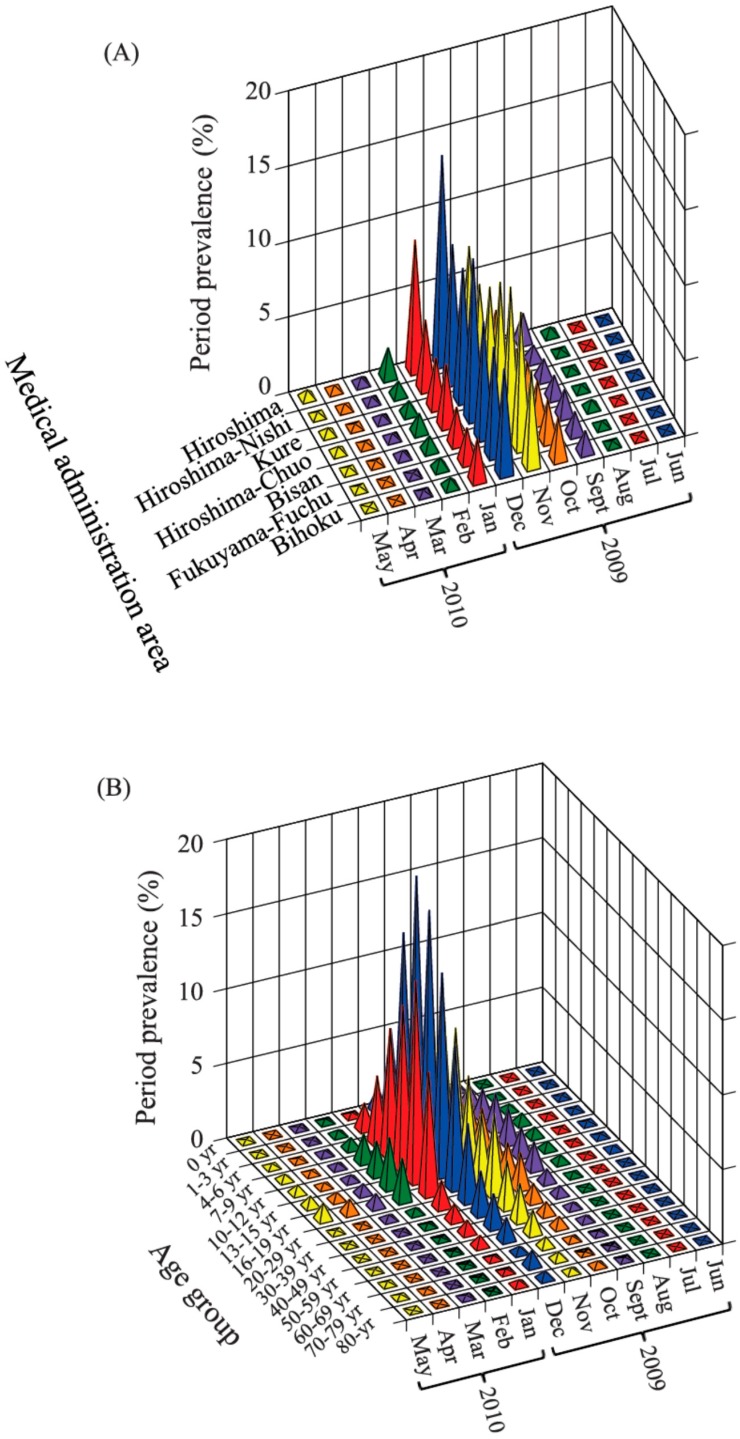
Period prevalence (%) from June 2009 to May 2010. Period prevalence is shown by: area (**A**); and age (**B**).

From June 2009 to May 2010, the period prevalence peaked in November in the Hiroshima-Nishi, Hiroshima, Kure, Hiroshima-Chuo, and Bihoku medical administration areas (from 6.9% to 13.8%), with Hiroshima (13.8%) and Hiroshima-Chuo (10.6%) displaying particularly high values. However, it peaked in December in Bisan and Fukuyama-Fuchu, and then decreased rapidly in January in all medical administration areas (Figure 2).

Age-specific period prevalence of participants aged 4–19 years and ≥70 peaked in November (from 0.5% to 13.6%), and that of those aged 1–3 years and 20–69 years peaked in December (from 0.7% to 7.2%). The period prevalence for both males (10,429/82,738; 12.6%) and females (10,251/93,375; 11.0%) peaked in November.

### 3.4. Overall Cumulative Incidence of Influenza, OR of Cumulative Incidence and VE Based on Vaccination Status

A total of 169,105 responses were available to estimate Influenza vaccine effectiveness and its cumulative incidence, 122,529 for the participants aged 0–15 years, and 46,576 for those aged 16 and over. As shown in Table 3, cumulative influenza incidence was significantly lower in the vaccinated group (18.9%) as compared with the non-vaccinated group (33.7%), with an OR of non-vaccinated group to vaccinated group for cumulative incidence of influenza A(H1N1)pdm09 and VE of 2.18 (95% CI: 2.13–2.23; *p* < 0.001) and 43.9% (95% CI: 42.8–44.9; *p* < 0.001), respectively. In the 1–3 years and 4–6 years groups, the ORs for cumulative incidences were 3.52 (95% CI: 3.25–3.81; *p* < 0.001) and 3.40 (95% CI: 3.25–3.56; *p* < 0.001), respectively. VE was 64.2% (95% CI: 61.6–66.6; *p* < 0.001) and 55.8% (95% CI: 54.3–57.2; *p* < 0.001) for the two groups, respectively. Compared to other age groups, these groups exhibited strong associations between vaccination and cumulative incidence. However, there was almost no association between vaccination and cumulative incidence in the 16–19 years age group (OR of cumulative incidence, 1.02; 95% CI: 0.95–1.10; *p* = 0.5214). In the 0-year groups, cumulative incidence was higher in the vaccinated groups compared to the non-vaccinated ones (OR, 0.35; 95% CI: 0.17–0.71; *p* = 0.0038). OR for cumulative incidence was from 1.59 to 2.68 (all *p* < 0.001) and VE was 28.0%–60.2% in the groups from 7–9 years to 50–59 years, except for 16–19 years.

In all medical administration areas, cumulative incidence was significantly higher in the non-vaccinated groups than in the vaccinated groups (*p* < 0.001). ORs in all the areas were ≥1.9.

### 3.5. Correlation between Cumulative Incidence and Vaccination Rate

Table 4 shows the correlation coefficients of area-specific vaccination rates of every age group and the area-specific overall cumulative incidence.

Our results indicate that medical administration areas with high vaccination rates for the 4–6 years and 10–12 years groups had significantly lower overall cumulative incidence. Correlation coefficients were −0.81 (*p* = 0.0266) for the 4–6 years group and −0.83 (*p* = 0.0212) for the 10–12 years group. However, the above correlation could not be detected in the rest of the groups.

### 3.6. Factors Associated with Influenza Infection

Table 5 presents the results of the multivariate analyses of the association between infection with influenza and studied parameters. We found that the results related to gender, medical administration area, age group, and helpful information were similar.

Overall, males were more susceptible to infection than females in the 0–15 years (adjusted OR 1.13; *p* < 0.001).

As for age groups, OR for cumulative incidence was significantly lower in the 0 year and 1–3 years groups as compared to the 4–6 years group (*p* < 0.001). However, it was significantly higher in the 7–9 years, 10–12 years, and 13–15 years groups (*p* < 0.001). ORs for cumulative incidence were significantly higher in the 16–19 years and 20–29 years groups compared to the 30–39 years group (*p* < 0.001). Furthermore, OR of cumulative incidence was significantly lower in the participants aged 40 years or over as compared to that in the 30–39 years group (*p* < 0.001).

As for medical administration area, Kure (*p* < 0.001) and Bihoku (*p* < 0.05) had significantly lower OR for cumulative incidence compared to Hiroshima. Except Hiroshima-Chuo, all medical administration areas had also significantly lower OR than Hiroshima in those aged 16 and over (Bihoku *p* < 0.05, all others *p* < 0.001). With the exception of Hiroshima-Chuo, in all the participants, Hiroshima-Nishi, Kure, Bisan, Fukuyama-Fuchu, and Bihoku had a significantly lower OR of cumulative incidence compared to Hiroshima (Hiroshima-Nishi *p* < 0.05, Bisan and Bihoku *p* < 0.01, all others *p* < 0.001).

As for preventative action, participants who were vaccinated had a low OR of cumulative incidence in both the 0–15 years participants (adjusted OR 0.55; *p* < 0.001) and all the participants (adjusted OR 0.62; *p* < 0.001). Participants aged 16 and over had a high OR of cumulative incidence (*p* < 0.001), except those who observed preventive measures such as “gargling and hand hygiene”.

### 3.7. Expansion of Infection

**Figure 3:**
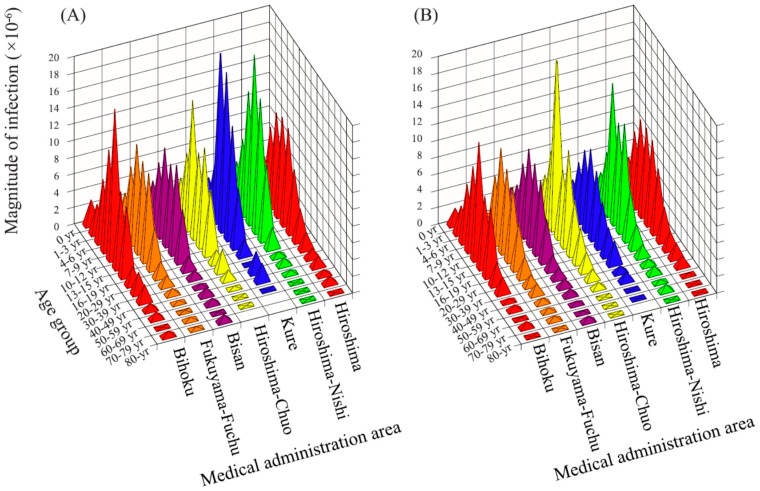
The expansion of infection. The expansion of infection is shown by age group and by medical administration area: male (**A**); and female (**B**).

The expansion of infection per group is shown in Figure 3. The expansion of infection was higher in the 7–9, 10–12, and 13–15 years age groups in every medical administration area and gender.

**Figure 4:**
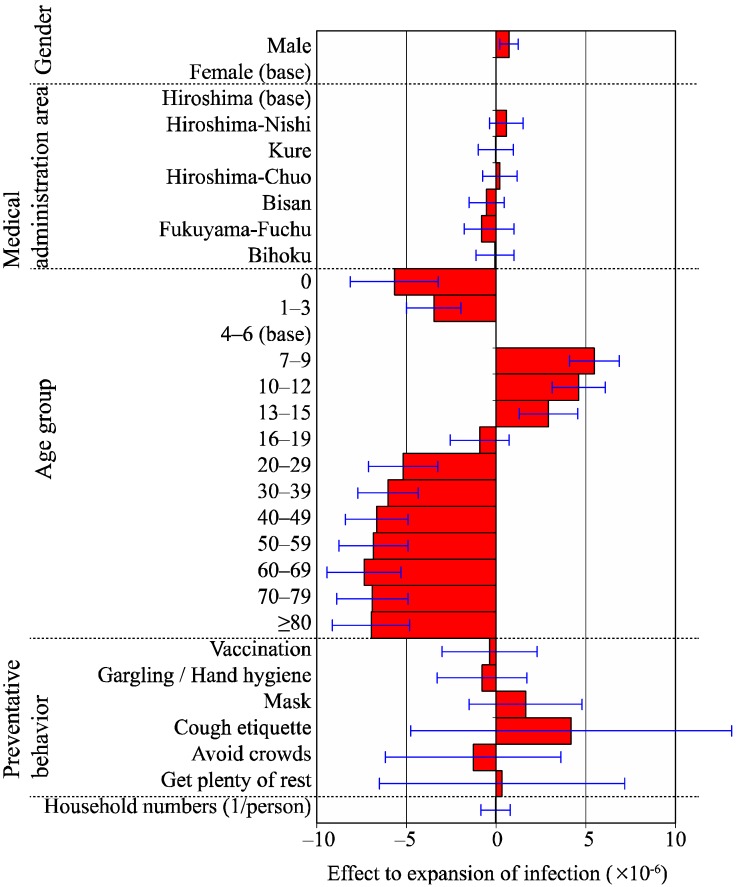
Effect of each factor to the expansion of infection. Estimated coefficient of each factor by multivariate linear regression (which means effect to expansion of infection) was shown as bar chart. The expansion of infection was higher in the male group (*p* < 0.05) as compared with the female group. It was also higher in the 7–9 years age group, followed by the 10–12 years age group and the 13–15 years age group as compared with the base group (4–6 years age group; *p* < 0.05).

The results of the multivariate analysis on the association between the expansion of infection and each factor are displayed in Figure 4. The coefficient of determination was 0.85. A significant difference in the expansion of infection was found between genders; being higher in the male group (*p* < 0.05). The expansion of infection was the highest in the 7–9 years age group, indicating a value that is 5.49 × 10^−6^ higher than that in the base group (4–6 years age group). It was followed by the 10–12 years age group and the 13–15 years age group, showing values that were higher by a factor of 4.62 × 10^−6^ and 2.92 × 10^−6^ than the base group, respectively (*p* < 0.05 for both).

When the effect of the expansion of the infection factor is positive, the person with the factor has a high tendency of spreading infection. The baseline group was defined as gender “Female”, medical administration area “Hiroshima”, and age group “4–6 years”. The effect of the expansion of the infection of household members correlates to a decrease or increase in the expansion of the infection at an increasing rate of one person per household.

## 4. Discussion

In this study, we analyzed data from a retrospective non-randomized cohort study on the influenza A(H1N1)pdm09 infection carried out in 176,113 residents of Hiroshima Prefecture. The results show that vaccinations during the influenza A(H1N1)pdm09 effectively reduced the cumulative incidence. In particular, vaccination for children was found to be effective in lowering the overall cumulative incidence in all medical administration areas.

Cumulative incidence was higher in the non-vaccinated groups than in the vaccinated groups in all of the medical administration areas during the pandemic. As for the age groups, the effectiveness of the seasonal H1N1 influenza vaccine among individuals has been previously reported [12,17,18,19,20]. In particular, this study revealed that vaccination had a strong preventative effect in people aged 1–3 years, 4–6 years, 20–29 years, and 30–39 years. The effect of vaccination in elderly populations appeared to be small, but since cumulative incidence in elderly people was low, the effect of vaccination cannot be directly inferred from the results. The same conclusion can be drawn for the 0 year age group. Furthermore, no children under six months were vaccinated. It might have had an effect on the VE. Although the 16–19 years age group exhibited high period prevalence from the onset of this pandemic, this group was given a low priority for vaccination. The VE was also low in this age group (16–19 years), but the precise explanation for this is unknown. In Japan, most high school students (16–18 years old) were vaccinated against influenza A(H1N1)pdm09 before university entrance exams around February. A student with confirmed influenza A(H1N1)pdm09 infection is likely not to be allowed to pass entrance exams. Therefore, some of the high school students might have been vaccinated after being infected by influenza A(H1N1)pdm09, thus reducing the vaccine effectiveness. These results may indicate that when the influenza vaccine is administered after the influenza A(H1N1)pdm09 epidemic peak, the vaccine effectiveness is reduced. In general, the effectiveness of vaccination would be expected to vary by age group based on the age-associated lifestyle habits, and by regional characteristics, such as population density and age structure. However, an overall OR of cumulative incidence of more than 2.0 and the large reduction in cumulative incidence seen with vaccination in the 4–6 years, 7–9 years, and 10–12 years groups indicate that vaccination strongly influenced the incidence of the 2009 influenza pandemic.

This study suggests that during the 2009 influenza pandemic, vaccinating schoolchildren was effective in creating a level of herd immunity in all of the medical administration areas. In the USA, influenza vaccinations have been reported to improve school attendance rates [21], and vaccinating children for influenza has been found to induce herd immunity in elderly populations [22]. Our findings support a possible sizable effect of schoolchildren (elementary to junior high school) on the spread of infection to the whole population. Since school-age populations can amplify seasonal influenza epidemics, Japan administered preventative vaccinations to schoolchildren from 1962 to 1994 as a public health measure to prevent epidemics from spreading throughout the community [10]. When this practice was halted, vaccination became voluntary. However, excess mortality from influenza was lower in Japan during the 1970s and 1980s when schoolchildren were being vaccinated, and increased when the practice was stopped in the 1990s [9]. A previous study reported that discontinuation of vaccination of schoolchildren was responsible for the increase in influenza-associated deaths among young children in the 1990s [23]. Influenza vaccines have also been reported to be cost-effective [24]. Using a mathematical epidemiological model, Nishiura et al. [25] found that during the H1N1 outbreak, infections may have spread among individuals aged 20 years and above. When making decisions on vaccination priority, not only high-risk populations should be targeted, but also populations with a potentially higher potential for transmission. Creating a system that can quickly and strongly recommend the vaccination of populations that have characteristics similar to the younger groups in this study, that is, populations who lack immunity to pandemic influenza and people who live in group settings, could be an effective method of prevention.

We examined the effect of each group on the period prevalence by defining the probability of transmission when infected persons had contact with susceptible persons as the expansion of infection. The expansion of infection estimated in this study was based on the multi-type epidemic model [16]. Although this enables a simulation based on real period prevalence by reflecting the action pattern by group, each parameter is difficult to estimate in the multi-type epidemic model using actual period prevalence data because of a large number of parameters to be estimated. Other models have been used to estimate the basic reproduction number per group in some studies. However, only a few studies, which targeted fewer groups, such as children versus adults, were able to estimate using real data [25,26]. Thus, to our best knowledge, no detailed study that divides the population by age or gender has been previously conducted. In our estimation, the expansion of infection was higher in the 7–15 years age group. These results could suggest that those groups could potentially make a large impact on the spread of infection. Previous studies reported that children play major roles in the spread of influenza within families or schools [27,28,29,30]. High contact frequency among schoolchildren can be considered as a causative factor of this phenomenon [31,32,33].

The results of the estimation of the expansion of infection per group indicate the group responsible for the spread of infection. The group with high expansion of infection should be targeted for operational policies. The recommended policy is school closure based on the results of this study that schoolchildren contribute to the spread of infection. In fact, the efficacy of school closure in the influenza A(H1N1)pdm09 period was reported in previous studies [34,35].

Two analyses regarding preventative action were performed in this study. The first analysis was an association between infection with influenza A(H1N1)pdm09 and preventative action. Participants aged 16 years and above who carefully observed preventative measures, such as gargling and hand hygiene, had a low probability of contracting influenza. Similarly, those in the 0–15 years age group and the combined analysis of all participants who were vaccinated were likely to resist against the influenza A(H1N1)pdm09. On the other hand, vaccination does not require knowledge of a skill and it can prevent children from contracting the influenza A(H1N1)pdm09 virus. The second analysis was the association between the expansion of infection (which could be an indirect indication of the power of transmission) and preventative action. No preventative action had a significant impact on controlling the spread of infection. Our results are inconsistent with previous reports that demonstrated the efficacy of preventative actions such as hand hygiene [14,36]. These differences might be explained by the difference in study designs. Our study was not an interventional study. Therefore, not all the participants observed preventive measures against influenza A(H1N1)pdm09 infection. However, the previous two studies were randomized controlled trials where participants in the intervention groups are likely to comply with preventive measures.

This study has several limitations. Firstly, we cannot show any causation from the results because this is a survey, but we took time relation into consideration to investigate the timing of infection. Secondly, data from this study were collected for practical purposes related to future influenza pandemics preparedness, and the number of younger (0–15 years) participants was higher than that of the elderly. However, our questionnaire survey covered all age groups and areas. Thirdly, the number of participants who reported having been infected with the influenza A(H1N1)pdm09 virus may be incorrect, since the diagnosis was based on answers from a questionnaire, but we distinguished whether they thought that they have been infected with the influenza A(H1N1)pdm09 virus and whether they were diagnosed with the influenza A(H1N1)pdm09 infection. Fourthly, we assumed no difference in the likelihood of infection in our model because nobody had antibody against influenza A(H1N1)pdm09 [37], but we did not take into consideration other biological characteristics. In addition, regarding the assumption that all infected persons would be cured by the following month based on the infection period of influenza, we created a model based on the monthly period prevalence of influenza even though the infectious state of influenza changes on a daily basis.

Fifthly, this study relied on voluntary response samples, with participants not being randomly selected. As a result, children (0–15) were oversampled (71.8% of the participants). Therefore, our surveys may be vulnerable to selection bias. However, the later concerns are mitigated by the fact that the sample size was deemed appropriate (*n* = 176,113) and the recruitment covered all areas and generations. People were likely to participate in this survey because the study was conducted by the Regional Health Care Council of Hiroshima Prefecture, which represents the Japanese government. Therefore, the effect of the bias seems to be small as compared with regular questionnaire surveys conducted in Japan.

Finally, this study contains recall bias. However, participants who were infected with influenza may remember clearly their preventative actions.

## 5. Conclusions

After the influenza A(H1N1)pdm09 outbreak, the Regional Health Care Council of Hiroshima Prefecture conducted a large survey of 176,113 people to address influenza outbreak preparedness. There are important findings that emerged from this study. Firstly, the vaccination of children created a level of herd immunity against influenza among the greater community. When an influenza pandemic occurs and vaccine supplies are limited, vaccinations should not only be provided to populations at high risk of mortality but also to groups that may spread the infection, with the aim of reducing the scale of the outbreak and lowering the overall risk of mortality. Secondly, schoolchildren have the high power to transmit the infection to others that we evaluate as the expansion of infection. Evaluating the dynamics of various infections by using expansion of infection as an index is considered to be helpful for devising infection-preventative measures in the future.

## Figures and Tables

**Table 1:** General characteristics of the participants.

Gender	Male	Female	Total
	*n* (%)	*n* (%)	*n* (%)
Total	82,738 (47.0)	93,375 (53.0)	176,113 (100.0)
Medical administration area			
Hiroshima	56,696 (32.2)	60,524 (34.4)	117,220 (66.6)
Hiroshima-Nishi	2962 (1.7)	2628 (1.5)	5590 (3.2)
Kure	3079 (1.7)	3849 (2.2)	6928 (3.9)
Hiroshima-Chuo	3149 (1.8)	3584 (2.0)	6733 (3.8)
Bisan	4721 (2.7)	5972 (3.4)	10,693 (6.1)
Fukuyama-Fuchu	10,129 (5.8)	14,504 (8.2)	24,633 (14.0)
Bihoku	1193 (0.7)	1687 (1.0)	2880 (1.6)
Other	809 (0.5)	627 (0.4)	1436 (0.8)
Age groups			
0 year	437 (0.2)	387 (0.2)	824 (0.5)
1–3 years	9230 (5.2)	8506 (4.8)	17,736 (10.1)
4–6 years	19,012 (10.8)	18,116 (10.3)	37,128 (21.1)
7–9 years ^†^	12,631 (7.2)	12,605 (7.2)	25,236 (14.3)
10–12 years ^††^	12,814 (7.3)	13,485 (7.7)	26,299 (14.9)
13–15 years ^†††^	9,174 (5.2)	10,092 (5.7)	19,266 (10.9)
16–19 years	12,741 (7.2)	11,812 (6.7)	24,553 (13.9)
20–29 years	932 (0.5)	4094 (2.3)	5026 (2.9)
30–39 years	1246 (0.7)	4082 (2.3)	5328 (3.0)
40–49 years	1302 (0.7)	3634 (2.1)	4936 (2.8)
50–59 years	1464 (0.8)	3847 (2.2)	5311 (3.0)
60–69 years	937 (0.5)	1552 (0.9)	2489 (1.4)
70–79 years	579 (0.3)	811 (0.5)	1390 (0.8)
≥80 years	239 (0.1)	352 (0.2)	591 (0.3)

^†^ Low grade of elementary school students; ^††^ High grade of elementary school students; ^†††^ Junior high school students.

**Table 2:** Vaccination rates.

	Total	Number of Vaccinated	Vaccination Rate (%)
Overall	157,084	57,320	36.5
By age group (*p* < 0.0001)			
0 year	812	85	10.5
1–3 years	17,220	9277	53.9
4–6 years	34,728	17,134	49.3
7–9 years	22,166	7759	35.0
10–12 years	22,309	5345	24.0
13–15 years	15,381	3076	20.0
16–19 years	20,647	6885	33.3
20–29 years	4706	1270	27.0
30–39 years	5047	1621	32.1
40–49 years	4682	1308	27.9
50–59 years	5082	1385	27.3
60–69 years	2387	989	41.4
70–79 years	1344	793	59.0
≥80 years	573	393	68.6
By medical administration area (*p* < 0.0001)			
Hiroshima-Nishi	5210	2015	38.7
Hiroshima	102,518	34,074	33.2
Kure	6539	2784	42.6
Hiroshima-Chuo	6277	2557	40.7
Bisan	10,152	4454	43.9
Fukuyama-Fuchu	23,630	10,108	42.8
Bihoku	2758	1328	48.2
By gender (*p* < 0.0001)			
Men	72,748	27,052	37.2
Women	84,336	30,268	35.9

**Table 3:** Overall cumulative incidence of influenza, OR of cumulative incidence and VE based on vaccination status (from November 2009 to May 2010).

	Number of Participants *n* (%)	Cumulative Incidence *n*/*N* (%)		Odds Ratio (OR)				Vaccine Effectiveness (VE; %)			
		Vaccinated Group	Non-Vaccinated Group	OR	95% Confidence Interval		*p* Value	VE (%)	95% Confidence Interval		*p* Value
Overall	153,308 (100.0)	10,855/57,320 (18.9)	32,379/95,988 (33.7)	2.18	2.13	2.23	<0.0001	43.9	42.8	44.9	<0.0001
**By Age Group (*p* < 0.0001) ^†^**											
0 year	755 (0.5)	10/85 (11.8)	31/690 (4.5)	0.35	0.17	0.71	0.0038	−161.9	−414.9	−33.2	0.0038
1–3 years	16,865 (11.0)	958/9277 (10.3)	2188/7588 (28.8)	3.52	3.25	3.81	<0.0001	64.2	61.6	66.6	<0.0001
4–6 years	34,016 (22.2)	3602/17,134 (21.0)	8025/16,882 (47.5)	3.40	3.25	3.56	<0.0001	55.8	54.3	57.2	<0.0001
7–9 years	21,728 (14.2)	2188/7759 (28.2)	6840/13,969 (49.0)	2.44	2.30	2.59	<0.0001	42.4	40.1	44.6	<0.0001
10–12 years	21,814 (14.2)	1526/5345 (28.6)	6931/16,469 (42.1)	1.82	1.70	1.94	<0.0001	32.2	29.0	35.2	<0.0001
13–15 years	14,990 (9.8)	753/3076 (24.5)	4049/11,914 (34.0)	1.59	1.45	1.74	<0.0001	28.0	23.0	32.6	<0.0001
16–19 years	19,962 (13.0)	1544/6885 (22.4)	2984/13,077 (22.8)	1.02	0.95	1.10	0.5214	1.7	−3.7	6.9	0.5214
20–29 years	4643 (3.0)	102/1270 (8.0)	565/3373 (16.8)	2.30	1.86	2.86	<0.0001	52.1	41.4	60.8	<0.0001
30–39 years	4969 (3.2)	63/1621 (3.9)	327/3348 (9.8)	2.68	2.05	3.49	<0.0001	60.2	48.2	69.4	<0.0001
40–49 years	4601 (3.0)	45/1308 (3.4)	244/3293 (7.4)	2.25	1.64	3.08	<0.0001	53.6	36.6	66.0	<0.0001
50–59 years	4950 (3.2)	32/1385 (2.3)	176/3565 (4.9)	2.20	1.52	3.17	<0.0001	53.2	32.1	67.7	<0.0001
60–69 years	2237 (1.5)	19/989 (1.9)	13/1248 (1.0)	0.54	0.27	1.06	0.0724	−84.4	−271.6	8.5	0.0723
70–79 years	1239 (0.8)	9/793 (1.1)	5/446 (1.1)	0.99	0.34	2.84	0.9816	−1.2	−200.2	65.9	0.9816
≥80 years	519 (0.3)	4/393 (1.0)	1/126 (0.8)	0.78	0.10	6.29	0.8140	−28.2	−1036.9	85.5	0.8138
**By Medical Administration Area (*p* = 0.0371) ^†^**											
Hiroshima-Nishi	5118(3.3)	269/2015 (13.3)	762/3103 (24.6)	2.11	1.82	2.45	<0.0001	45.6	38.3	52.1	<0.0001
Hiroshima	100,116 (65.3)	7232/34,074 (21.2)	23,995/66,042 (36.3)	2.12	2.06	2.18	<0.0001	41.6	40.2	42.9	<0.0001
Kure	6359(4.1)	432/2784 (15.5)	950/3575 (26.6)	1.97	1.74	2.23	<0.0001	41.6	35.3	47.3	<0.0001
Hiroshima-Chuo	6158(4.0)	501/2557 (19.6)	1141/3601 (31.7)	1.90	1.69	2.14	<0.0001	38.2	32.2	43.6	<0.0001
Bisan	9935(6.5)	716/4454 (16.1)	1676/5481 (30.6)	2.30	2.09	2.53	<0.0001	47.4	43.2	51.4	<0.0001
Fukuyama-Fuchu	22,926(15.0)	1554/10,108 (15.4)	3499/12,818 (27.3)	2.07	1.94	2.21	<0.0001	43.7	40.6	46.6	<0.0001
Bihoku	2696(1.8)	151/1328 (11.4)	356/1368 (26.0)	2.74	2.24	3.35	<0.0001	56.3	48.0	63.3	<0.0001
**By Gender (*p* = 0.8469) ^†^**											
Men	70,849(46.2)	5603/27,052 (20.7)	15,913/43,797 (36.3)	2.18	2.11	2.26	<0.0001	43.0	41.5	44.5	<0.0001
Women	82,459(53.8)	5252/30,268 (17.4)	16,466/52,191 (17.4)	2.20	2.12	2.27	<0.0001	45.0	43.5	46.5	<0.0001

^†^ *p* values were assessed by Breslow-Day test.

**Table 4:** Correlation between cumulative incidence of influenza and vaccination coverage rate.

	Correlation Coefficient	*p* Value
0 year	−0.24684	0.5936
1–3 years	−0.24976	0.5891
4–6 years	−0.81175	0.0266
7–9 years	−0.46113	0.2977
10–12 years	−0.82873	0.0212
13–15 years	−0.44746	0.3141
16–19 years	−0.3034	0.5083
20–29 years	−0.06394	0.8917
30–39 years	−0.36538	0.4203
40–49 years	−0.33015	0.4696
50–59 years	−0.44907	0.3121
≥60 years	−0.51402	0.2379

**Table 5:** Factors associated with influenza infection ^†^.

Item	0–15 Years (*n* = 125,477)			≥16 Years (*n* = 49,231)			All Participants (*n* = 174,708)		
	AOR	95% CI	*p* Value	AOR	95% CI	*p* Value	AOR	95% CI	*p* Value
Gender									
Male	1.16	1.14–1.19	<0.001 ***	1.03	0.98–1.08	0.3070	1.13	1.11–1.16	<0.001 ***
Female	1.00			1.00			1.00		
Medical administration area									
Hiroshima	1.00			1.00			1.00		
Hiroshima-Nishi	1.00	0.92–1.08	0.9085	0.79	0.70–0.88	<0.001 ***	0.92	0.86–0.98	0.0148 *
Kure	0.75	0.70–0.81	<0.001 ***	0.80	0.72–0.88	<0.001 ***	0.77	0.73–0.82	<0.001 ***
Hiroshima-Chuo	1.07	0.99–1.14	0.0730	0.90	0.82–1.00	0.0515	1.02	0.96–1.08	0.5631
Bisan	1.02	0.96–1.08	0.5127	0.67	0.61–0.74	<0.001 ***	0.93	0.88–0.97	0.0021 **
Fukuyama-Fuchu	0.99	0.95–1.02	0.4583	0.65	0.60–0.70	<0.001 ***	0.90	0.87–0.94	<0.001 ***
Bihoku	0.88	0.78–0.98	0.0192 *	0.76	0.61–0.93	0.01 *	0.86	0.78–0.95	0.0032 **
Other	0.95	0.73–1.24	0.7079	0.60	0.49–0.72	<0.001 ***	0.73	0.62–0.84	<0.001 ***
Age group									
0 year	0.10	0.07–0.13	<0.001 ***				0.10	0.07–0.13	<0.001 ***
1–3 years	0.46	0.44–0.48	<0.001 ***				0.46	0.44–0.48	<0.001 ***
4–6 years	1.00						1.00		
7–9 years	1.35	1.30–1.40	<0.001 ***				1.34	1.30–1.39	<0.001 ***
10–12 years	1.25	1.21–1.29	<0.001 ***				1.26	1.22–1.30	<0.001 ***
13–15 years	1.12	1.08–1.17	<0.001 ***				1.14	1.10–1.19	<0.001 ***
16–19 years				5.04	4.55–5.60	<0.001 ***	0.80	0.77–0.83	<0.001 ***
20–29 years				2.01	1.78–2.27	<0.001 ***	0.30	0.28–0.33	<0.001 ***
30–39 years				1.00			0.15	0.14–0.17	<0.001 ***
40–49 years				0.74	0.64–0.85	<0.001 ***	0.11	0.10–0.13	<0.001 ***
50–59 years				0.52	0.44–0.61	<0.001 ***	0.08	0.07–0.09	<0.001 ***
60–69 years				0.21	0.15–0.29	<0.001 ***	0.03	0.02–0.04	<0.001 ***
70–79 years				0.23	0.15–0.34	<0.001 ***	0.03	0.02–0.05	<0.001 ***
≥80 years				0.14	0.05–0.28	<0.001 ***	0.02	0.01–0.04	<0.001 ***
Helpful information									
Television	0.82	0.79–0.85	<0.001 ***	0.74	0.68–0.81	<0.001 ***	0.81	0.78–0.83	<0.001 ***
Newspapers	0.87	0.84–0.90	<0.001 ***	0.80	0.72–0.88	<0.001 ***	0.86	0.83–0.89	<0.001 ***
Pamphlets	0.96	0.90–1.01	0.1399	1.04	0.88–1.22	0.6696	0.97	0.92–1.02	0.2761
Internet	0.93	0.89–0.98	0.0034 **	0.79	0.69–0.89	0.0002 ***	0.90	0.87–0.95	<0.001 ***
Preventative behavior									
Vaccination	0.55	0.53–0.56	<0.001 ***	1.10	1.04–1.16	0.0008 ***	0.62	0.61–0.64	<0.001 ***
Gargling/Hand hygiene	1.19	1.16–1.23	<0.001 ***	0.91	0.87–0.96	0.0005 ***	1.11	1.08–1.14	<0.001 ***
Mask	1.53	1.50–1.57	<0.001 ***	1.89	1.80–1.99	<0.001 ***	1.61	1.58–1.65	<0.001 ***
Cough etiquette	1.33	1.28–1.39	<0.001 ***	1.44	1.30–1.60	<0.001 ***	1.34	1.29–1.40	<0.001 ***
Avoid crowds	1.16	1.18–1.19	<0.001 ***	1.37	1.26–1.49	<0.001 ***	1.19	1.16–1.23	<0.001 ***
Get plenty of rest	1.00	0.97–1.04	0.9334	1.26	1.16–1.36	<0.001 ***	1.04	1.01–1.08	0.0081 **

0–15 years survey: *R*^2^ = 0.06, model *p* < 0.001. ≥16 years survey: *R*^2^ = 0.14, model *p* < 0.001. All participants: *R*^2^ = 0.10, model *p* < 0.001. AOR: adjusted odds ratio; CI: confidence interval. * *p* < 0.05; ** *p* < 0.01; *** *p* < 0.001; ^†^ Baseline groups: gender “female”, medical administration area “Hiroshima”, age group “4–6 years” (for all participants and those aged 0–15 years) or “30–39 years” (for participants ≥16 years), “no information was helpful”, and “did not pay attention to anything”.

## Supplementary Materials

The following are available online at www.mdpi.com/1660-4601/13/10/1017/s1, Supplementary File: Sample items used to assess factors related to the expansion of infection and prevention of the influenza A(H1N1)pdm09.
