# Supplementary material for: High Vaccination Coverage among Children during Influenza A(H1N1)pdm09 as a Potential Factor of Herd Immunity
Source: Int J Environ Res Public Health. 2016 Oct 17;13(10):1017. doi: 10.3390/ijerph13101017 (PMC5086756; doi:10.3390/ijerph13101017)
Supplement: Supplementary file 1 [file ijerph-13-01017-s001.pdf]

Supplementary Materials: High Vaccination Coverage among Children during Influenza A(H1N1)pdm09 as a Potential Factor of Herd Immunity

Toshihiko Matsuoka, Tomoki Sato, Tomoyuki Akita, Jiturou Yanagida, Hiroki Ohge, Masao Kuwabara and Junko Tanaka

Supplementary File. Sample items used to assess factors related to the expansion of infection and prevention of the influenza A(H1N1)pdm09. The survey was applied to participants aged 0–15 years and those aged 16 years and over.

Anonymous questionnaire

I. Demographic information

1 Where do you live?

□ Hiroshima-city □ Kure-city □ Takehara-city □ Mihara-city □ Onomichi-city
□ Fukuyama-city □ Fuchu-city □ Miyoshi-city □ Shoubara-city □ Otake-city
□ Higashimiroshima-city □ Hatsukaichi-city □ Akitakata-city □ Etajina-city
□ Fuchu-town □ Kaita-town □ Kumano-town □ Saka-town □ Akiota-town
□ Kitahiroshima-town □ Osakikamijima-town □ Sera-town □ Jinsekikogen-town
□ Other areas( )

2 How old is your child?/How old are you?

( ) years old

□ 15–19 □ 20–29 □ 30–39 □ 40–49 □ 50–59 □ 60–69 □ 70–79 □ 80 and over

3 What is the gender of your child?/What is your gender?

□ Male □ Female

II. Information related to attitude, knowledge and contraction of the influenza A(H1N1)pdm09

4 What was the most helpful information about influenza A(H1N1)pdm09?

□ TV □ Pandemics papers □ Brochures □ Internet
□ others()

5 Have you thought that you/your child suffered from influenza A(H1N1)pdm09?

□ Yes □ No
→ If "yes":

5-1 what kind of action did you take?

□ I consulted a family medical institution.
□ I consulted a medical institution but not a family medical institution.
□ In consultation with "fever consultation center" such as public health centers, I consulted a specific medical institution.
□ In consultation with "fever consultation center" such as public health centers, I/my child underwent medical treatment at home.
□ I/my child did not have any medical examination.

6 Did a doctor confirm your/your child's influenza A(H1N1)pdm09 infection from June, 2009 to May, 2010?

□ Yes No

→ If "Yes":

6-1 When did you /your child have a diagnosis of influenza A(H1N1)pdm09?

□ June 2009 □ July 2009 □ August 2009 □ September 2009 □ October 2009 □ November 2009
□ December 2009 □ January 2010 □ February 2010 □ March 2010 □ April 2010 □ May 2010

7 Was your child absent from school (including kindergarten) because of influenza A(H1N1)pdm09?/Were you absent from factories/companies or school because of influenza A(H1N1)pdm09?

□ Yes No

→ If "Yes":

7-1 How many days did you/your child take a rest?

( ) days

7-2 What is the reason for you/your child to be absent from school/work?

□ Because my child suffered from influenza A(H1N1)pdm09
□ Because my family suffered from influenza A(H1N1)pdm09
□ Because I was told to take a rest from a school (including kindergarten)/work.
□ Because a school (including kindergarten) stopped
□ Because I was anxious about suffering from influenza A(H1N1)pdm09
□ others( )

8 How many people are living together in your family?

Household size including children: ( ) people
Number of household members who had a diagnosis of influenza A(H1N1)pdm09: ( ) people

8-1 Who had an accurate diagnosis of influenza A(H1N1)pdm09 in your household?

□ Child □ Grandfather □ Grandmother □ Father □ Mother
□ Brothers and Sisters □ Others( )

9 Which preventative action did you take against influenza A(H1N1)pdm09? (Please choose up to two answers)

□ Vaccination
□ Thorough gargling/hand hygiene
□ Use of a face mask
□ Cough etiquette
□ Avoidance of being in crowds
□ Getting plenty of rest
□ I did not take any preventative action
□ Others( )

10 Was your child vaccinated against influenza A(H1N1)pdm09?

Were you vaccinated against influenza A(H1N1)pdm09?

□ Yes □ No

11. What is the reason for being vaccinated? (Please choose up to two answers)

□ Because it was recommended by doctors
□ Because it was recommended to a family
□ Because I did not want to be absent from work/school
□ Because I was in a previously vaccinated group
□ Because it was recommended by media
□ Because it was free
□ Because I thought that it was effective for the prevention

12 For persons avoiding vaccination. What is the reason for not being vaccinated? (Please choose up to two answers)

□ Because I hate being vaccinated
□ Because I have already suffered from influenza A(H1N1)pdm09
□ Because I thought that a seasonal flu shot was enough
□ Because I cannot expect an effect
□ Because I was running out of time
□ I hoped, but there were not sufficient vaccine.
□ Because I was charged a fee (Because it is not free of charge???)

13 What were uneasy things (source of anxiety) regarding influenza A(H1N1)pdm09? (Please choose up to two answers)

□ Consequences of influenza A(H1N1)pdm09 on my/my child health and life
□ I cannot go to school (including kindergarten)/work
□ There having been too much information (information was complicated)
□ Go in a crowd
□ There were no uneasy things (anxiety)

14 Do you have any suggestion/opinion reading influenza A(H1N1)pdm09? If yes, please write your suggestion/opinion in the box below.

Thank you for your cooperation.
